# 加速康复外科在中国大陆胸外科临床现状——基于胸外科医生及护士调查分析

**DOI:** 10.3779/j.issn.1009-3419.2017.03.03

**Published:** 2017-03-20

**Authors:** 娜 杜, 成林 郭, 梅 杨, 艳丽 戢, 维 王, 洁 李, 川 李, 伦旭 刘, 国卫 车

**Affiliations:** 610041 成都，四川大学华西医院胸外科 Department of Thoracic Surgery, West China Hospital, Sichuan University, Chengdu 610041, China

**Keywords:** 加速康复外科, 胸外科, 调查问卷, 中国大陆, Enhanced recovery after surgery, Thoracic surgery, Survey Questionnaire, Mainland China

## Abstract

**背景与目的:**

虽然加速康复外科（enhanced recovery after surgery, ERAS）理念近年来已逐渐被外科医生所熟悉和应用于临床实践中，但目前关于我国大陆胸外科医师对ERAS理念的认知和应用现状如何仍不清楚。本研究基于对参会胸外科医生和护士进行ERAS相关问题的问卷调查结果，分析加速康复外科在胸外科的应用现状和面临的困难。

**方法:**

对参与第一届胸科ERAS华西论坛代表回复的773份有效问卷进行分析，问卷内容主要包括两部分：一是被调查人单位情况及个人基本情况；二是加速康复外科相关的10个问题。

**结果:**

①ERAS的临床应用现状为理念大于实践，69.6%的医生和58.7%的护士认同此观点；88.5%的医生和85.7%护士均认为ERAS理念适用于所有外科。②ERAS临床应用依从性差的主要原因是方案不成熟、无共识和规范（55.6%的医生和69.1%的护士）。③ERAS临床实施的最佳团队组合是外科为主的学科协作及医护一体（62.1%的医生和70.7%的护士）。④73.7%的医生和81.9%的护士认为ERAS的评价标准应为：平均住院日、患者感受和社会满意度进行综合评价。

**结论:**

加速康复外科在胸外科应用现状仍然是理念大于实践，主要原因是缺乏临床可用的规范和方案。

## Abstract (en)

**Background and objective:**

Though the concept of enhanced recovery after surgery (ERAS) has been progressively known by the surgeons and applied clinically, the current status of its cognition among thoracic surgeons and application in thoracic surgery is still unknown. Based on the analysis of a survey of thoracic surgeons and nurses on chest ERAS during a national conference, we aimed to analyze the status and difficulties of the application of ERAS in thoracic surgery.

**Methods:**

A total of 773 questionnaires were collected during the first West China chest ERAS Forum and analyzed. The content of the questionnaire can be divided into two parts, including the respondents' institute and personal information, 10 questions on ERAS.

**Results:**

(1) Current status of clinical application of ERAS is the concept rather than the practice: 69.6% of the surgeons and 58.7% of the nurses agreed with this view; in addition, 88.5% of the doctors and 85.7% of the nurses believed that the concept of ERAS may be applicable to every branches of surgery; (2) 55.6% of the doctors and 69.1% of the nurses believed that the reason of poor clinical application of ERAS included no mature procedure, lack of consensus and specifications; (3) The best team for the clinical practice of ERAS should be based on surgeon-centered multidisciplinary cooperation and integration of medical care: 62.1% of the surgeons and 70.7% of nurses agreed with this view; (4) 73.7% of the surgeons and 81.9% of the nurses agreed that mean hospital stay, patients' experience in hospital and social satisfaction should be the evaluation standard of ERAS practice.

**Conclusion:**

The application of ERAS in thoracic surgery is still the concept rather than the practice. The reason included the lack of clinical applicable specifications and scheme.

加速康复外科（enhanced recovery after surgery, ERAS）理念近年来已逐渐被外科医生所熟悉和应用于临床实践中。ERAS的内涵是减少创伤对机体的应激反应，促进机能快速康复；外延体现在临床上降低并发症发生率并缩短住院时间^[1]^。加速康复起源于欧洲和北美洲^[2]^，主要体现在术前和术后的管理流程优化，强调住院日缩短和费用降低。而随后对ERAS理念的认识逐渐转变为需要多学科协作与医护一体化管理。中国大陆地区对于ERAS理念的认识也逐渐增加，推广和应用得较好的医院主要集中在少数几个国家级三级甲等医院，而全国大多数省市级医院的胸外科对其认识和开展的较少。对大多数胸外科医师对ERAS理念的认知和应用现状如何不清楚，并无相关文献报道。对这些问题的不了解，将导致ERAS理念在各大医院的应用和推广难以奏效。因而我们通过问卷调查的形式，对来自我国大陆部分31个省级行政区域，包括22个省、5个自治区、4个直辖市医院的胸外科各级医师和护士对ERAS的认知，在其医院开展情况和发展方向等问题进行了调查和分析，以期为ERAS理念在临床的推广和应用，规范和完善实施ERAS的具体操作流程和制订提供参考。

## 1 对象与方法

### 1.1 调查对象

对2016年11月25日-26日参加并注册的第一届胸科ERAS华西论坛810名人员进行调查，收到有效问卷773人份。773名医护人员来自大陆（22个省，5个自治区和4个直辖市）（香港、澳门及台湾省无代表）370家医院，不同区域参与调查人数情况见图 1。

**1:**
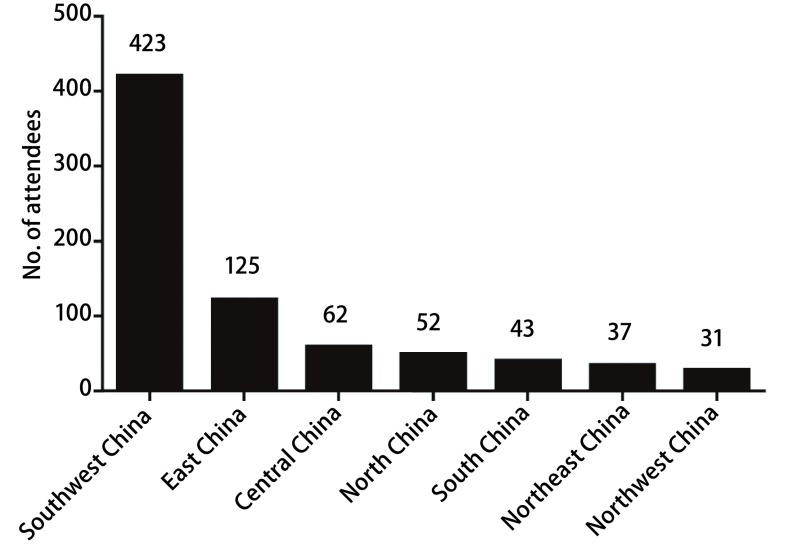
中国大陆不同区域参与调查人数 Number of attendees from different regions of Mainland China who participated the survey

### 1.2 方法

采用问卷调查法。本次问卷主要包括两个部分：第一部分：①受访对象的基本情况，包括年龄、性别、职业、职称、职务和单位所在省（市）；②所在医院胸外科基本情况，包括科室床位数、科室年手术量和胸腔镜手术比例。第二部分包括10个问题，内容主要涉及所在医院胸外科对加速康复理念的认识和应用，包括：①ERAS的应用范围；②ERAS的应用现状；③ERAS的评价标准；④ERAS方案实施的最佳团队组合；⑤ERAS应用中依从性差的主要因素；⑥ERAS理念在临床上的应用；⑦个人对临床上应用ERAS理念的认识；⑧ERAS方案实施的最佳模式；⑨ERAS方案实施的推动途径；⑩ERAS会议的主要内容。问卷由会议工作人员统一提供并支持二维码扫描电子答题，当场填写完成后统一提交到管理系统。

### 1.3 统计学分析

对所有有效问卷进行描述性分析和本实验采用SPSS 21.0软件对数据进行统计学处理分析，计量资料用均数±标准差（Mean±SD）表示，组间比较采用*t*检验，计数资料采用*χ*^2^检验，*P*≤0.05为差异有统计学意义。

## 2 结果

### 2.1 参会人员基本情况及科室年胸腔镜手术量分析

370所医院均具备开展胸腔镜手术的条件，139所（37.57%, 139/370）胸腔镜手术量＞60%，141所（38.11%, 141/370）介于30%-60%之间，90所（24.32%, 90/370）＜30%。

773份调查问卷中，包括医生514人（66.5%, 514/773），中位年龄39岁，护士259人（33.5%, 259/773），中位年龄34岁；高级职称324人，中级职称238人，初级职称211人；部级医院94人，部队医院62人，省级医院270人，市级医院296人，县级医院51人；正（副）院长7人，正（副）主任或护士长310人，无行政职务者456人。

### 2.2 加速康复外科应用现状分析

69.6%的医生和58.7%的护士认为ERAS的应用现状为理念大于实践（图 2A）；88.5%的医生和85.7%护士认为ERAS理念适用于所有外科（图 2B）；59.3%的医生和50.2%的护士认为所在医院只有部分科室将ERAS理念应用于临床（图 2C）；45.3%的医生和33.3%的护士在临床上将ERAS理念应用部分患者，而40.2%的护士在临床实践中将ERAS理念应用于所有患者（图 2D）。

**2:**
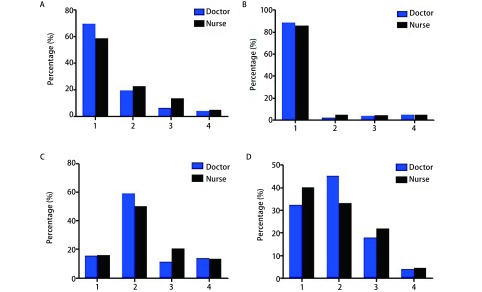
ERAS临床应用现状。A：ERAS应用现状（1.理念大于实践；2.国外优于国内；3.普外科用得好；4.胃肠外科用得好）；B：ERAS应用范围（1.外科都该用；2.普外科可以用；3.单病种用比较好；4.特定手术方式）；C：ERAS理念在临床上的应用（1.所有科室都用；2.部分科室用；3.外科用得好；4.医院关注）；D：个人对临床上应用ERAS理念的认识（1.所有患者都用；2.部分患者用；3.部分手术用；4.不用）。 Current status of the clinical application of ERAS. A: Current status of ERAS (1. Concept more than practice; 2. Abroad prior to China; 3. Mainly in General Surgery Department; 4. Applicated well in Gastrointestinal Surgery); B: Scope of ERAS (1. Applicable in all branches of surgery; 2. Could be applied in General Surgery Department; 3. Good to be applied in single diseases; 4. Applied in specific operations); C: Application of ERAS (1. Applied in all wards; 2. Applied in some of the department; 3. Applied well in the Department of Surgery; 4. Administrator of the hospital paid attention to ERAS); D: Personal application of ERAS (1. Applied in all patients; 2. Applied in some of the patients; 3. Applied in some of the operations; 4. Not applied). ERAS: enhanced recovery after surgery.

### 2.3 加速康复外科临床应用困难及实现途径

55.6%的医生和69.1%的护士认为方案不成熟、无共识和规范以及医患安全无保障是ERAS应用中依从性差的主要因素（图 3A）；62.1%的医生和70.7%的护士认为学科整合、以外科为主的联合以及医护一体化均为ERAS实施的最佳团队组合（图 3B）；38.1%的医生和57.5%的护士认为多学科协作、以外科为主的多模式和外科制定方案均是ERAS实施的最佳模式（图 3C）；42.8%的医生和31.7%的护士认为协会发布规范是推动ERAS实施的最佳途径，而44.8%的护士认为医院行政推动才是ERAS实施的最佳途径（图 3D）。

**3:**
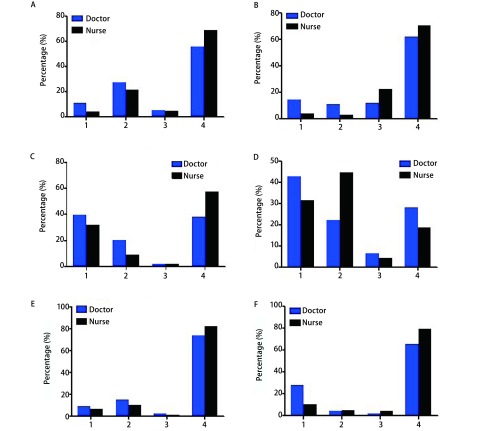
ERAS临床应用困难、实现途径、评价标准及会议内容。A：ERAS依从性差的原因分析（1.方案不成熟；2.无共识和规范；3.医患安全无保障；4.以上全是）；B：ERAS实施的最佳团队（1.学科整合；2.外科为主，联合；3.医护一体；4.以上全是）；C：ERAS实施的最佳方式（1.多学科协作；2.外科为主，多模式；3.外科制订方案；4.以上全是）；D：ERAS实施的推动途径（1.协会发布规范；2.医院行政推动；3.科室自发处理；4.个体化执行）；E：ERAS的评价标准（1.平均住院日；2.患者感受；3.社会满意度；4.以上全是）；F：ERAS会议内容（1.规范与共识；2.项目与实施；3.现状与进展；4.以上全是）。 Difficulties in the clinical application of ERAS, way of implementation, the standard of assessment and conference content. A: the causes of poor compliance of ERAS (1. Absence of mature ERAS scheme; 2. Lack of concensus and standard; 3. Unguaranteed clinical safety for the patients and surgeons; 4. All above); B: the best team on the implementation of ERAS (1. Integration of the disciplines; 2. Multi-disciplinary co-operation conducted by surgery; 3. Integration between surgeons and nurses; 4. All above); C: The best way to ERAS implementation (1. Multi-disciplinary co-operation; 2. Muti-modality conducted by surgery; 3. Practice scheme formulated by surgery; 4. All above); D: The best way to ERAS implementation (1. Standard released by the Society; 2. Promote the application through administrative means of the hospital; 3. Spontaneous practice in each department. 4. All above); E: Evaluation standard of ERAS (1. Mean hospital stay; 2. Patients'experience; 3. Social satisfaction; 4. All above); F: ERAS conference contents (1. Concensus and Standard; 2. Program and Practice; 3. Current status and Progression; 4. All above).

### 2.4 加速康复外科评价标准及会议内容

73.7%的医生和81.9%的护士认为ERAS的评价标准：平均住院日、患者感受和社会满意度进行综合评价（图 3E）；65.6%的医生和79.5%的护士认为ERAS现状与进展、临床研究项目和临床应用经验交流、规范与共识应该是ERAS会议的主要内容（图 3F）。

## 3 讨论

ERAS理念率先由丹麦学者Kehlet^[3]^于1997年首次提出，经过近20年的发展，取得了令人瞩目的成绩，已成为英国和加拿大两国政府主导的临床路径。2007年黎介寿院士^[4]^将ERAS理念引入我国。虽然ERAS理念已从最初只应用于结直肠手术逐步推广到临床各外科领域，但推广过程并不顺利，不同层次医院的应用情况也参差不齐。在欧洲的一项调查发现，欧洲国家中只有1/3的医院在应用ERAS，传统习惯和理念是阻碍ERAS推广和应用的主要阻碍^[5]^。我们的调查表明，我国大部分医护人员能正确认识到ERAS理念不应局限于某一外科领域，而应该应用于所有外科，但大多数医护人员仍认为我国胸外科ERAS开展的现状为理念大于实践。这表明不仅欧洲运用ERAS遇阻，我国的情况同样不容乐观，仍面临大量的研究成果局限于临床试验、难以推广应用的困境。同时，在对临床上哪些患者应该应用ERAS理念上，医生和护士的观念亦存在差异，大多数医生认为临床上只有部分患者适用，而护士认为应该将ERAS方案应用于所有患者。

ERAS在临床应用中需要多模式或多学科协作完成^[1]^。我们的调查结果表明我国大部分胸外科医护人员能接受这一观点，认为学科整合、外科为主的联合以及医护一体化均为ERAS实施的最佳团队组合。虽然ERAS在胸外科应用的重要价值已逐渐被国内外的医疗中心所重视，但在实施过程中，作为实施主体的医护人员依从性仍然较差。我们的调查结果表明，大多数胸外科医师所在的医院只有部分科室运用ERAS理念。方案不成熟、缺乏共识和规范以及医患关系无保障均是导致依从性差的主要原因。这也提示我们改变传统观念，完善方案，制定ERAS实施过程中的指南和规范，制定保障医患安全的措施是推动ERAS顺利实施的保障。

当前各个学科将缩短住院时间和降低术后并发症作为评价ERAS方案是否可行的主要标准^[6]^。但开展ERAS的临床意义绝不仅仅是缩短住院时间和降低术后并发症。例如，我们不能为了缩短住院时间，而勉强地、不安全地让患者出院。人是生物、心理和社会的综合体，现代医学已从以疾病为中心的生物医学模式转换为以人为中心的生物-心理-社会医学模式。所以综合评价住院时间、患者和社会的满意度才是评价ERAS方案是否可行的标准。我们的调查结果表明，我国大多数胸外科医护人员能准确认识到不能采用单一的标准来评价ERAS方案是否可行。

外科、麻醉和护理等多学科的协作和配合是ERAS顺利实施的保障^[7]^。我们的调查结果表明，我国大多数胸外科医护人员能认识到多学科协作、外科为主的多模式和外科制定方案均为ERAS实施过程中的最佳方案。ERAS实施过程中的特殊性决定了医院行政管理部门和为实施过程制定规范化流程的重要性。目前国内外已经发布了部分有关ERAS的共识与指南，例如，成立于2010年的欧洲ERAS协会，目前已召开多次国际大会，制定了胰十二指肠切除、结直肠切除和胃切除术等ERAS专家共识与指南^[8-11]^；我国于2015年成立ERAS协作组，发布了《结直肠手术应用加速康复外科中国专家共识（2015版）》^[12]^。然而国内外目前仍缺乏胸外科专科的ERAS共识与指南。我们的调查表明，绝大多数胸外科医护人员赞成协会发布规范和医院行政推动是ERAS实施的主要推动途径。

我国ERAS研究起步较晚，直到2015年，才在南京召开了第一届ERAS全国大会。鉴于我国举办ERAS会议尚缺乏经验，哪些内容是参会者想在参会过程中学习的以及以何种方式将大会办得更亲民是主办方非常关注的问题。我们的调查结果表明，规范与共识、项目与实施以及ERAS的现状和进展是参会者主要关注的议题。该结果为我们明年举办第二届胸科ERAS华西论坛提供了参考。

我国ERAS研究与应用已进入一个快速发展的上升期，但在推广和应用方面仍面临许多问题和挑战，在我国医院的大多数胸外科的应用现状仍局限于理论阶段。其原因主要是方案不成熟、无共识和规范以及医患安全无保障。针对以上问题，制定胸外科专科的ERAS临床规范与指南以及医院行政干预在促进ERAS在胸外科应用方面非常重要。
